# Risk of Migraine after Traumatic Brain Injury and Effects of Injury Management Levels and Treatment Modalities: A Nationwide Population-Based Cohort Study in Taiwan

**DOI:** 10.3390/jcm12041530

**Published:** 2023-02-15

**Authors:** Mei-Hui Chen, Yueh-Feng Sung, Wu-Chien Chien, Chi-Hsiang Chung, Jeng-Wen Chen

**Affiliations:** 1 Department of Medical Education and Research, Far-Eastern Memorial Hospital, New Taipei City 220, Taiwan; 2 Department of Neurology, Tri-Service General Hospital, National Defense Medical Center, Taipei 114, Taiwan; 3 Department of Medical Research, Tri-Service General Hospital, National Defense Medical Center, Taipei 114, Taiwan; 4 School of Public Health, National Defense Medical Center, Taipei 114, Taiwan; 5 Department of Otolaryngology–Head and Neck Surgery, Cardinal Tien Hospital, School of Medicine, Fu Jen Catholic University, New Taipei City 231, Taiwan; 6 Department of Otolaryngology–Head and Neck Surgery, National Taiwan University Hospital, Taipei 100, Taiwan; 7 Master Program of Big Data in Biomedicine, School of Medicine, Fu Jen Catholic University, New Taipei City 242, Taiwan; 8 Department of Medical Education and Research, Cardinal Tien Hospital, New Taipei City 231, Taiwan

**Keywords:** traumatic brain injury, migraine, epidemiology, headache, treatment modalities

## Abstract

Traumatic brain injury (TBI) causes several long-term disabilities, particularly headaches. An association between TBI and subsequent migraine has been reported. However, few longitudinal studies have explained the link between migraine and TBI. Moreover, the modifying effects of treatment remain unknown. This retrospective cohort study used records from Taiwan’s Longitudinal Health Insurance Database 2005 to evaluate the risk of migraine among patients with TBI and to determine the effects of different treatment modalities. Initially, 187,906 patients, aged ≥ 18 years, who were diagnosed as TBI in 2000, were identified. In total, 151,098 patients with TBI and 604,394 patients without TBI were matched at a 1:4 ratio according to baseline variables during the same observation period. At the end of follow-up, 541 (0.36%) and 1491 (0.23%) patients in the TBI and non-TBI groups, respectively, developed migraine. The TBI group exhibited a higher risk of migraine than the non-TBI group (adjusted HR: 1.484). Major trauma (Injury Severity Score, ISS ≥ 16) was associated with a higher migraine risk than minor trauma (ISS < 16) (adjusted HR: 1.670). However, migraine risk did not differ significantly after surgery or occupational/physical therapy. These findings highlight the importance of long-term follow-up after TBI onset and the need to investigate the underlying pathophysiological link between TBI and subsequent migraine.

## 1. Introduction

Traumatic brain injury (TBI) is defined as the disruption of brain function or other evidence of brain pathology caused by an external physical force [1]. TBI results in more deaths and disabilities than any other traumatic insult worldwide [2]. The estimated annual occurrence of TBI varies widely, ranging from 2.5 million in the European Union to 3.5 million in the USA [3]. Due to increased road traffic, the incidence is typically higher in developing countries [4,5,6]. For example, in India, TBI causes an estimated 1 million disabilities annually and accounts for one fatality every three minutes [3].

TBI causes not only short-term impairment, but also persistent and even life-long consequences [7]. Negative outcomes following TBI include persistent postconcussive symptoms (PCS) [8], neurodegenerative disorders [9], psychological disorders, including post-traumatic stress disorder (PTSD) [10], psychiatric sequelae [11], sleep disturbances [12], autonomic dysfunction [13], and suboptimal health-related quality of life, particularly in women [14]. Of these, headache is one of the most common postconcussive disorders [15]. Moreover, among the various etiologies of headache, TBI has been suggested to be a risk factor for migraine [15,16,17].

Migraine is a primary headache with a 1-year prevalence of up to 15% in the general population [18]. It is characterized by recurrent attacks of headache with a range of accompanying symptoms. By contrast, post-traumatic headache (PTH) is a secondary headache attributed to trauma or injury to the head or neck. PTH can be classified as acute if it develops within seven days after a TBI and resolves within three months, or it can be classified as chronic if it persists for more than three months [19]. The most common PTH patterns resemble two primary headache disorders, migraine or probable migraine and tension-type headaches. However, migraine is more prevalent [13].

At present, effective treatment for TBI is lacking [20,21]. Nonetheless, standard medical and surgical interventions play a significant role in the acute management for TBI. Surgical intervention is usually warranted when a significant mass effect occurs that results from a hematoma or a contusion with a significant volume of blood [22]. Following this, some of the most severe TBI patients can survive with impaired neurological function [23]. Moreover, because of the long-term effects of TBI, early follow-up and further rehabilitation are essential to facilitate recovery [24,25].

Globally, mild TBI accounts for more than 80% of the reported TBI cases [2,26,27,28]. Most studies have revealed that mild TBI is associated with a higher risk of PTH than moderate-to-severe TBI [29,30,31]. However, whether the risk of migraine development after TBI decreases with an increase in the severity of TBI remains unknown. Therefore, we hypothesized that the risk of migraine development increases after TBI and is associated with the different severity of TBI. This study assessed the risk of subsequent migraine among TBI patients and determined the modifying effects of different management levels and treatment modalities.

## 2. Materials and Methods

### 2.1. Data Sourses

This retrospective cohort study was conducted using data from the Longitudinal Health Insurance Database 2005 (LHID2005), a subset of Taiwan’s National Health Insurance (NHI) Research Database (NHIRD). This study was reviewed and approved by the Institutional Review Boards of Cardinal Tien Hospital (CTH-110-3-5-039) and Tri-Service General Hospital (B-110-45). The requirement of written informed consent from participants was waived for this analysis of data from a deidentified database.

### 2.2. Study Design and Sampled Participants

**Figure 1:**
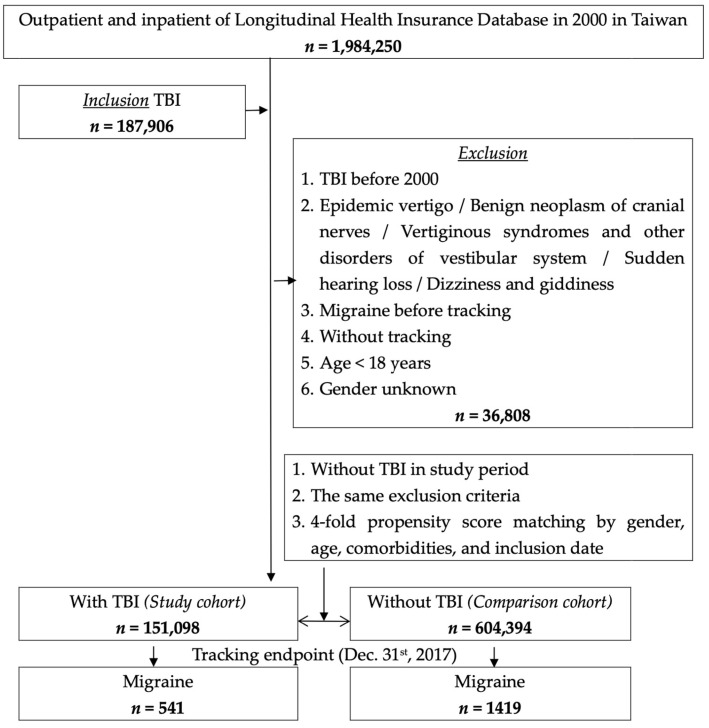
Flow diagram of study sample selection.

Of the 1,984,250 patients with outpatient or inpatient records in the LHID2005 claims data in 2000 (Figure 1), we identified 187,906 patients diagnosed as having TBI, according to the International Classification of Diseases, Ninth Revision, Clinical Modification (*ICD-9-CM*; 2000–2015) diagnostic codes, with the diagnosis being made at least thrice in the outpatient department (OPD), once in the emergency department, or once on admission. Patients who received a TBI diagnosis in any specialty were included. Patients who were younger than 18 years or who had a history of TBI or other diseases that may cause vertigo or dizziness before the index date were excluded. Moreover, patients diagnosed as having migraine before the index date and those with incomplete demographic data were excluded. In total, 151,098 patients with newly diagnosed TBI were enrolled into the TBI cohort. For each patient in the TBI cohort, four patients without a history of TBI were selected from the remaining records by propensity score matching, according to sex, age, comorbidities, and index date (non-TBI cohort). The exclusion criteria were the same for both the cohorts. The matched non-TBI cohort included 604,394 patients, and the date of the records used for their selection served as the index date. The diagnostic codes for the inclusion and exclusion variables are presented in Appendix Table A1.

### 2.3. Outcome Measurement

Both the cohorts were followed up from the index date to the date of migraine onset, withdrawal from the NHI program, or the end of follow-up. For outcome measurement, migraine was defined using the *ICD-9-CM* diagnostic code 346 and the *ICD-10-CM* diagnostic code G43. Patients who received a diagnosis of migraine from a neurologist or an otolaryngologist were enrolled into this study. The cumulative incidence of migraine was estimated according to the TBI status using the Kaplan–Meier method, and differences between the cumulative incidence rates were compared using a log-rank test. Moreover, Cox proportional hazards models were used to compute the crude and adjusted hazard ratios (HRs) and 95% confidence intervals (CIs) for migraine between the TBI and non-TBI groups and between different TBI subgroups. Injury severity scores (ISS) [32,33] were used to assess the severity of injury and to predict mortality, morbidity, and length of hospital stay. The ISS ranges from 1 to 75. As per the NHI program in Taiwan, ISS ≥ 16 denotes the presence of major trauma and a catastrophic illness. Patients with any defined catastrophic illness can benefit from copayment exemptions.

### 2.4. Potential Confounders

We adjusted for the following confounders: sex, age/age group, geographic location in Taiwan, urbanization level, insurance premium, season, and level of care. Individuals with or without the comorbidities listed in Table 1, on or before the index date, were stratified by the aforementioned confounders for comparison.

### 2.5. Subgourp Analysis

Subgroup analysis was performed according to TBI treatments to determine the effect of the interventions on migraine risk. Brain surgery, involving microvascular decompression, craniotomy or cranioplasty, ventriculostomy, hematoma removal, endarterectomy, and bypass surgery, were included as surgical treatment. Moreover, chronic rehabilitation programs included occupational therapy (OT) and physical therapy (PT).

### 2.6. Statistical Analysis

All statistical analyses were performed using IBM SPSS Statistics version 22 (IBM, Armonk, NY, USA). The chi-squared test and Student’s *t*-test were used to assess the distributions of categorical and continuous variables, respectively. Multivariate Cox proportional hazards regression analysis was conducted to determine the risk of migraine. The results are presented as HRs and 95% CIs. The differences in the risk of migraine between the TBI and non-TBI cohorts were assessed using the Kaplan–Meier method and log-rank tests. A two-tailed *p* value of <0.05 was considered significant.

## 3. Results

### 3.1. Baseline Characteristics

The baseline characteristics of the TBI and non-TBI cohorts are presented in Table 1. The mean age of the TBI cohort was 44.43 ± 18.55 years. No significant differences in sex, age, or comorbidities were noted between the TBI and non-TBI cohorts after propensity score matching. The average follow-up period was 10.75 and 10.88 years for the TBI and non-TBI cohorts, respectively (Table 2). Appendix Table A2 presents the characteristics of the TBI and non-TBI cohorts at the end of follow-up.

### 3.2. Kaplan–Meier Model for Assessing the Cumulative Risk of Migraine

**Figure 2:**
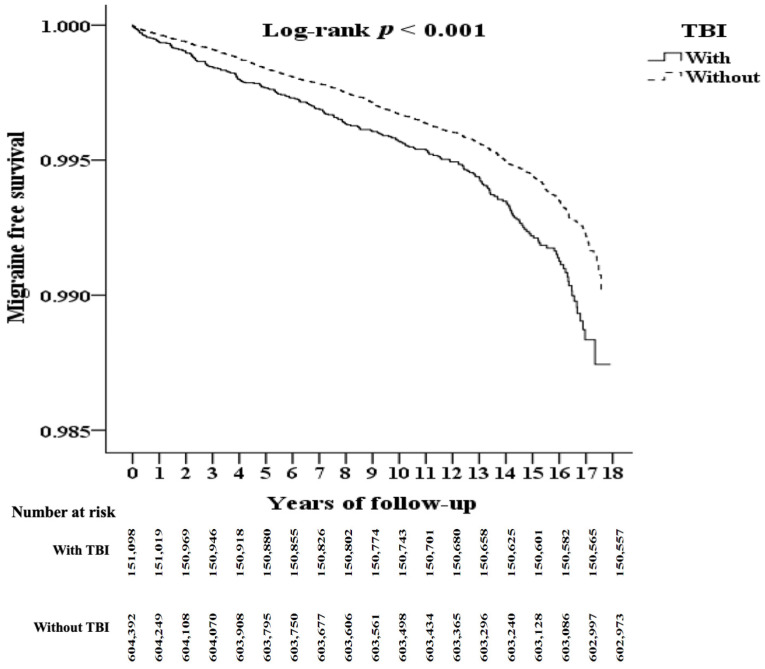
Kaplan–Meier analysis of cumulative risk of migraine stratified by TBI using the log-rank test.

At the end of follow-up, 541 (0.36%) of 151,098 TBI patients and 1419 (0.23%) of 604,394 non-TBI controls had developed migraine (*p* < 0.001). Kaplan–Meier analysis revealed that the cumulative risk of migraine significantly differed between the TBI and non-TBI cohorts over the 18-year follow-up period (log-rank test, *p* < 0.001, Figure 2).

### 3.3. HRs for Migraine in the TBI Cohort

AppendixTable A3 lists the factors that were associated with migraine by the end of follow-up in the Cox regression model. In the TBI cohort, the crude HR for migraine was 1.688 (95% CI: 1.454–2.006, *p* < 0.001). After adjustment for age, sex, comorbidities, insurance premium, geographic location, urbanization level, and level of care, the adjusted HR was 1.484 (95% CI: 1.276–1.724, *p* < 0.001). The TBI cohort exhibited a higher risk of migraine than the non-TBI cohort, as revealed by subgroup analyses stratified by sex, age group, insurance premium, comorbidities, urbanization level, geographic location, and level of care (Appendix Table A4).

### 3.4. HRs for Migraine Subtypes in the TBI Cohort

Table 3 presents the results of Cox regression analyses of migraine subtypes in the TBI cohort. No significant differences were noted between the risk of migraine with and without aura. Moreover, no significant differences were noted in the diagnoses made by otolaryngologists and neurologists.

### 3.5. HRs for TBI Subtypes

Table 4 presents the results of Cox regression analyses of TBI subtypes in the TBI cohort. Regarding the severity of injuries, the risk of migraine with ISS ≥ 16 was higher in the TBI cohort than the risk of migraine with ISS < 16 (adjusted HR: 1.670, 95% CI: 1.325–2.011, *p* < 0.001). Hospitalized patients exhibited a significantly higher risk of subsequent migraine than those visiting the OPD (adjusted HR: 1.557, 95% CI: 1.203–1.837, *p* < 0.001).

### 3.6. Effects of Treatment Modalities of TBI on Risk of Migraine

Table 4 presents the results of Cox regression analyses of treatment modalities in the TBI cohort. No significant differences were noted between the TBI subgroups with and without brain surgery. Similarly, no significant differences were noted between the TBI subgroups with and without OT/PT and pharmacological treatment. The percentage of participants who received OT/PT between those with and without brain surgery in the TBI cohort showed no significant difference between the two groups (Table 5). However, TBI patients who received brain surgery had a significantly longer duration and higher intensity (times) of OT/PT within one year of TBI occurrence (Table 6).

## 4. Discussion

In this study, the TBI cohort exhibited a higher risk of subsequent migraine than the propensity score–matched non-TBI cohort. The incidence of migraine following major trauma (ISS ≥ 16) was higher than that following minor trauma (ISS < 16) in the TBI cohort. Hospitalized TBI patients exhibited a higher risk of migraine than those who visited the OPD. Furthermore, surgery or OT/PT did not significantly reduce the risk of migraine. These results suggest that, in addition to providing acute surgical intervention and chronic rehabilitation, physicians should counsel TBI patients regarding adjuvant strategies to prevent subsequent migraine development.

### 4.1. Pathophysiological Links between Migraine and TBI

Whether trauma induces migraine or triggers a pre-existing susceptibility to migraine itself remains unclear. Several factors may be involved in the risk of migraine-type headache, including axonal injury, changes in cerebral autoregulation, and genetic stability [17,34,35,36]. For example, cellular injury following TBI increases the concentration of extracellular potassium, which can trigger neuronal depolarization and the release of neurotransmitters that promote the development of headaches [37]. Neuroinflammation may also play a role in brain injury [38,39], which is associated with repeated sports-associated TBI events [40,41,42], and headache is a part of its symptom spectrum [40]. Moreover, inflammation and other responses to injury can enhance neuronal excitability [43]. Hyperexcitability of trigeminal nerve branches mediates throbbing head pain in patients with migraine [10].

### 4.2. Effects of the Severity of TBI on the Risk of Migraine

In this population-based study of Taiwanese adults, the TBI cohort exhibited a 1.484-fold increased risk of migraine, which is in accordance with previous findings, suggesting TBI to be a risk factor for migraine [15,16,17]. Compared with TBI patients diagnosed in the OPD or emergency department, hospitalized TBI patients exhibited an increased risk of subsequent migraine. Similarly, compared with TBI patients with ISS < 16, those with ISS ≥ 16 exhibited an increased risk of subsequent migraine. These results suggest that patients with a higher severity of TBI exhibit a higher risk of migraine. However, these results contradict previous findings that mild TBI is associated with a higher risk of migraine [10,15,16,19,29,30]. These discrepant findings may be attributed to several factors. First, various criteria have been used to define the severity of TBI. These include the duration of loss of conscious [44], Glasgow Coma Scale score [5,36,45], and duration of post-traumatic amnesia (PTA). A recent study even identified more than 50 definitions for mild TBI [46]. These varying definitions may lead to differing results. Second, the inclusion criteria and sample selection processes were different in the studies. As the TBI group mainly includes patients with mild TBI, the literature largely includes samples with mild TBI and associated postconcussive disorder. Third, according to Do et al., sociodemographic differences, such as the absence of a third-party insurance program, are responsible for the discrepancies [47]. However, previous studies have not explained why migraine develops more frequently after mild TBI [15,16].

Some studies have assessed the occurrence, longitudinal course, associated factors, and characteristics of headache in more severe TBI patients. For example, one study revealed that patients who continued to experience headaches three months after TBI were more likely to exhibit slow continued recovery, particularly after a year of persistent headaches and particularly if their TBI was moderate or severe [17]. Another study revealed that patients with a history of moderate TBI had higher odds of reporting severe headaches (adjusted odds ratio: 3.89) and migraine-like features (adjusted odds ratio: 15.34) than those with subconcussive exposure, which was limited to mild TBI [44]. Furthermore, a study revealed that moderate and severe TBI can disrupt the blood–brain barrier and thus allow the migration of neutrophils from leaky blood vessels, resulting in neuroinflammation, which plays a key role in the pathophysiology of post-traumatic headache [39]. Thus, moderate or severe TBI may result in more injury and an increased risk of migraine.

We propose some possible explanations for these results. First, the follow-up times and methods for measuring tracking progress after TBI differed in the studies [48]. A recent study revealed that most patients improve within a few days to a few weeks; however, many patients continue to report PCS for months or years, even after very minor head injuries [49]. Walker et al. reported another type of headache course after severe TBI, which is known as delayed-onset headache, the symptoms of which do not manifest until after acute rehabilitation. In their study, the occurrence rate of delayed-onset headaches over the one-year period after discharge was 22% [50]. Second, measuring the progress and outcomes following neuropsychological rehabilitation for mild TBI is challenging because of the variability of baseline symptoms, the subjectivity of many common problems, and the lack of a reliable relationship between objective measures (such as neuropsychological tests and neuroimaging) and the subjective sense of progress or success [51]. Thus, studies with shorter follow-up times or difficulty in tracking may have underestimated the number of patients who developed migraine after moderate or severe TBI. Third, studies on the association between the severity of TBI and pain have reported mixed findings. In these studies, information was collected based on patient reports [30,44]. Hence, multiple factors, such as sampling bias [50], assessment methods, study types, and cultural and language backgrounds [52], can explain the discrepancies observed in the prevalence of post-traumatic headache in different studies. Patients with mild TBI have been reported to be more susceptible to perceiving pain and have a lower pain threshold [38]. Moreover, patients with more severe TBI may have difficulty in reporting or processing their symptoms because of memory disturbance, language deficit, and executive dysfunction. Thus, more reports of migraine may be observed after mild TBI than after moderate or severe TBI. A prospective controlled study assessing the risk of migraine in TBI patients is, therefore, warranted to confirm this association.

### 4.3. Effects of Treatment Modalities for TBI on the Risk of Migraine

To the best of our knowledge, this is the first study to examine the association between treatment modalities for TBI and the risk of migraine by using data from a nationwide population-based database. No significant difference was noted in the risk of migraine between patients undergoing brain surgery and those receiving OT/PT. However, evidence of the association between treatment modalities and the risk of migraine is still lacking. Further studies are warranted to assess the association between treatment modalities for TBI and the risk of migraine.

Many factors can affect recovery from TBI; these include injury characteristics, neuropathological findings, premorbid personality traits, and psychological characteristics [53]. Moreover, several studies have revealed that migraine-like headaches are linked to slow recovery [54,55,56]. Rehabilitation after brain injury can promote recovery through three main approaches: spontaneous improvement that can prevent complications in days to months, increase in neuroplasticity that can result in functional restitution, and compensative maximization of independence and quality of life [20].

Most approaches for treating post-traumatic headache with migraine-like features are derived from those effective in treating migraine headaches [49,57]. Nonpharmacological approaches involve lifestyle modifications, such as exercise, good sleep, hydration, and management of stress or events that can trigger migraine attacks. Managing anxiety may reduce ongoing symptoms [58]. Moreover, managing socioeconomic and family-related stressors plays a crucial role in managing the effects of persistent PCS [49,59]. Furthermore, pharmacological treatment [60], such as acute or preventive medications for primary headache disorders, is useful [19,25,61].

### 4.4. Strengths and Limitations of This Study

Our study has several important strengths. First, this longitudinal study involved a large cohort, providing sufficient power to detect associations and to adjust for a wide range of potential confounders. Second, the baseline characteristics, such as comorbidities, did not differ significantly, thereby decreasing the heterogeneity usually noted in a civilian study population. Third, the follow-up period in our study was quite long (>10 years). This may decrease the possibility of the under-identification of patients who developed migraine in a later period after TBI. Fourth, we not only demonstrated the prevalence of migraine among TBI patients, but we also described the relationship of migraine with the severity of brain injury and treatment modalities. Finally, to increase the validity of our findings, we only included patients who received a diagnosis of migraine from an otolaryngologist or a neurologist.

Our study also has several limitations. First, TBI and migraine were diagnosed based on ICD codes instead of using validated structural diagnostic instruments or the International Classification of Headache Disorders, 3rd edition codes. Moreover, detailed medical records, including OT/PT’s treatment intensity, were unavailable in the deidentified claims data. However, to improve the accuracy of our definition of migraine, we only used diagnoses made by otolaryngologists and neurologists. Additionally, we could not identify the severity of TBI based on *ICD-9-CM* codes. Hence, we used data on ISSs and management levels (e.g., treatment on OPD visits, emergent department visits, or hospitalization) to distinguish the severity of TBI. However, this may not accurately reflect the severity of TBI. Second, data on residual confounders, including genetic, physical, psychological, behavioral, and other socioenvironmental parameters related to different types of migraine, were not available in the NHIRD. However, adjusting for age and sex in the analysis may partially control for this factor. Furthermore, the baseline characteristics did not differ significantly in our study, which may reduce the heterogeneity. Third, despite being derived from a population-based study, our results may not be generalizable to other countries with populations with different ethnicities and backgrounds. Fourth, several studies have revealed that patients with a family history of headache are more likely to exhibit a migraine phenotype than those without this family history [51]. However, we could not assess the effect of family history in the claims database. Finally, because of the retrospective study design, we could not determine the causal relationship between TBI and migraine. Additional prospective trials are warranted to clarify the causal relationship between TBI and migraine and to determine the effects of treatment on the risk of migraine in TBI patients.

## 5. Conclusions

This study demonstrated that TBI was associated with a 1.484-fold increased risk of migraine. Moreover, among TBI patients, hospitalization and major trauma (ISS ≥ 16) were associated with 1.557-fold and 1.670-fold increased risks of migraine, respectively. No significant differences were noted between the treatment modalities after TBI. These findings highlight the importance of long-term follow-up after TBI and the need to further assess the underlying pathophysiological link between TBI and subsequent migraine.

## Figures and Tables

**Table 1:** Characteristics of the study population at baseline.

TBI	Total		With		Without		*p* Value
Variables	*n*	%	*n*	%	*n*	%	
**Total**	755,490		151,098	20.00	604,392	80.00	
**Sex**							0.999
Male	469,605	62.16	93,921	62.16	375,684	62.16	
Female	285,885	37.84	57,177	37.84	228,708	37.84	
**Age (years)**	44.45 ± 18.76		44.43 ± 18.55		44.46 ± 18.81		0.578
**Age group (yrs)**							0.999
18–29	221,875	29.37	44,375	29.37	177,500	29.37	
30–39	129,805	17.18	25,961	17.18	103,844	17.18	
40–49	132,020	17.47	26,404	17.47	105,616	17.47	
50–59	89,195	11.81	17,839	11.81	71,356	11.81	
≧60	182,595	24.17	36,519	24.17	146,076	24.17	
**Insured premium (NT$)**							<0.001
<15,840	739,707	97.91	147,919	97.90	591,788	97.91	
15,841–25,000	11,311	1.50	2471	1.64	8840	1.46	
>25,001	4472	0.59	708	0.47	3764	0.62	
**Hypertension**							0.656
Without	707,968	93.71	141,556	93.68	566,412	93.72	
With	47,522	6.29	9542	6.32	37,980	6.28	
**Diabetes mellitus**							0.693
Without	717,896	95.02	143,609	95.04	574,287	95.02	
With	37,594	4.98	7489	4.96	30,105	4.98	
**Depression**							0.164
Without	754,065	99.81	150,792	99.80	603,273	99.81	
With	1425	0.19	306	0.20	1119	0.19	
**Congestive Heart Failure**							0.511
Without	753,032	99.67	150,620	99.68	602,412	99.67	
With	2458	0.33	478	0.32	1980	0.33	
**Cerebrovascular accident**							0.708
Without	739,105	97.83	147,802	97.82	591,303	97.83	
With	16,385	2.17	3296	2.18	13,089	2.17	
**Chronic Obstructive Pulmonary Disease**							0.547
Without	745,876	98.73	149,199	98.74	596,677	98.72	
With	9614	1.27	1899	1.26	7715	1.28	
**Liver cirrhosis**							0.538
Without	740,388	98.32	148,119	98.34	592,269	98.32	
With	12,644	1.68	2501	1.66	10,143	1.68	
**Alcoholism**							0.751
Without	749,242	99.17	149,859	99.18	599,383	99.17	
With	6248	0.83	1239	0.82	5009	0.83	
**Chronic Kidney Disease**							0.903
Without	748,708	99.10	149,746	99.11	598,962	99.10	
With	6782	0.90	1352	0.89	5430	0.90	
**Osteoporosis**							0.617
Without	754,083	99.81	150,809	99.81	603,274	99.82	
With	1407	0.19	289	0.19	1118	0.18	
**Hyperlipidemia**							0.697
Without	751,341	99.45	150,258	99.44	601,083	99.45	
With	4149	0.55	840	0.56	3309	0.55	
**Autoimmune Disease**							0.527
Without	755,298	99.97	151,056	99.97	604,242	99.98	
With	192	0.03	42	0.03	150	0.02	
**Season**							0.999
Spring (Mar–May)	189,785	25.12	37,957	25.12	151,828	25.12	
Summer (Jun–Aug)	188,910	25.00	37,782	25.00	151,128	25.00	
Autumn (Sep–Nov)	190,670	25.24	38,134	25.24	152,536	25.24	
Winter (Dec–Feb)	186,125	24.64	37,225	24.64	148,900	24.64	
**Location**							<0.001
Northern Taiwan	295,631	39.13	40,025	26.49	255,606	42.29	
Central Taiwan	221,807	29.36	52,864	34.99	168,943	27.95	
Southern Taiwan	193,680	25.64	48,299	31.97	145,381	24.05	
Eastern Taiwan	41,169	5.45	9287	6.15	31,882	5.28	
Outlying islands	3203	0.42	623	0.41	2580	0.43	
**Urbanization level**							<0.001
1 (The highest)	247,547	32.77	32,824	21.72	214,723	35.53	
2	316,113	41.84	59,273	39.23	256,840	42.50	
3	70,152	9.29	20,006	13.24	50,146	8.30	
4 (The lowest)	121,678	16.11	38,995	25.81	82,683	13.68	
**Level of care**							<0.001
Hospital center	229,155	30.33	22,648	14.99	206,507	34.17	
Regional hospital	251,367	33.27	47,438	31.40	203,929	33.74	
District hospital	274,968	36.40	81,012	53.62	193,956	32.09	

**Table 2:** Comparison of years of follow-up and years to migraine onset in the TBI and non-TBI cohorts.

	Years of Follow-Up				Years to Migraine			
TBI	Min	Median	Max	Mean ± SD	Min	Median	Max	Mean ± SD
With	0.01	8.63	17.86	10.75 ± 8.42	0.02	6.18	17.49	7.02 ± 4.86
Without	0.01	8.86	17.93	10.88 ± 8.65	0.03	6.75	17.62	7.41 ± 5.03
Overall	0.01	8.79	17.93	10.85 ± 8.60	0.02	6.64	17.62	7.33 ± 5.00

**Table 3:** Assessment of factors associated with migraine subgroups using Cox regression analysis.

TBI	With vs. Without (Reference)			
Migraine Subgroup	Adjusted HR	95% CI	95% CI	*p* Value
**Overall**	1.484	1.276	1.724	<0.001
Migraine with aura	1.558	1.341	1.806	<0.001
Migraine without aura	1.464	1.260	1.709	<0.001
Diagnosis by otolaryngologist	1.395	1.201	1.638	<0.001
Diagnosis by neurologist	1.572	1.343	1.799	<0.001

Adjusted HR = adjusted hazard ratio (adjusted for the variables listed in Table A2); CI = confidence interval.

**Table 4:** Assessment of factors associated with the occurrence of migraine among different TBI subgroups using Cox regression analysis.

TBI Subgroup	Populations	Adjusted HR	95% CI		*p* Value	Adjusted HR	95% CI		*p* Value
**Without TBI**	604,392	Reference							
**With TBI**	151,098	1.484	1.276	1.724	<0.001				
OPD	45,330	1.251	1.081	1.459	<0.001	Reference			
ER	54,783	1.293	1.120	1.498	<0.001	1.060	0.878	1.365	0.124
ADM	50,985	1.915	1.648	2.222	<0.001	1.557	1.203	1.837	<0.001
**Without brain surgery**	84,456	1.495	1.311	1.739	<0.001	Reference *			
Without OT/PT	43,727	1.536	1.321	1.784	<0.001	Reference			
With OT/PT	40,729	1.504	1.293	1.742	<0.001	0.978	0.613	1.174	0.389
**With brain surgery**	66,642	1.469	1.265	1.708	<0.001	0.998	0.662	1.234	0.472
Without OT/PT	34,492	1.489	1.281	1.730	<0.001	0.965	0.604	1.136	0.427
With OT/PT	32,150	1.377	1.186	1.605	<0.001	0.893	0.588	1.025	0.433
**Without OT/PT**	78,219	1.487	1.313	1.768	<0.001	Reference			
**With OT/PT**	72,879	1.452	1.240	1.682	<0.001	0.983	0.624	1.199	0.397
**ISS < 16**	103,153	1.233	1.060	1.435	<0.001	Reference			
**ISS ≥ 16**	47,945	2.023	1.742	2.359	<0.001	1.670	1.325	2.011	<0.001
**Without pharmacological treatment**	31,296	1.480	1.271	1.719	<0.001	Reference			
**With pharmacological treatment**	119,802	1.485	1.279	1.727	<0.001	1.001	0.672	1.287	0.594

Adjusted HR = adjusted hazard ratio (adjusted for the variables listed in Table A3); CI = confidence interval. * Compared with those with brain surgery.

**Table 5:** Crosstab of brain surgery and OT/PT in the TBI cohort.

Brain Surgery	Total		With		Without		p *
Variables	*n*	%	*n*	%	*n*	%	
**Total**	151,098	100	66,642	44.11	84,456	55.89	
**OT/PT**							0.945
Without	78,219	51.77	34,492	51.76	43,727	51.77	
With	72,879	48.23	32,150	48.24	40,729	48.23	

* p: Chi-square test.

**Table 6:** Duration (months) and intensity (times) of OT/PT within one year of TBI occurrence.

Brain Surgery	Population	Duration (Months) Mean (SD)	*p* *	Intensity (Times) Mean (SD)	*p* *
With	32,150	11.14 (10.22)		7.4 (6.7)	
Without	40,729	9.86 (9.51)		6.7 (6.3)	
Overall	72,879	10.42 (9.85)	<0.001	7.0 (6.5)	<0.001

* *p*: independent *t*-test.

## Data Availability

The data presented in the study are available upon request from the corresponding author.

## Appendix A

**Table A1:** Diagnostic codes (*ICD-9-CM* and *ICD-10-CM* codes) for the inclusion and exclusion variables and NHI and ATC codes for medications.

Variables	Abbreviation	ICD-9-CM/ICD-10/NHI Code/ATC Code/Definition
**Study population:** Traumatic brain injury	TBI	310.2, 800–804, 850–854, 870–873, 905.0, 907.0, 950.1, 950.3, 950.91, V15.52; S01, S02, S06, T90
Minor cases	OPD	≧3 outpatient department visits
Emergent cases	ER	Emergency room visits
Severe cases	ADM	Admission visits
**Brain surgery**		Any of the following
Microvascular decompression		OP04.41, 83001B, 83030B, 83087B
Craniotomy/Cranioplasty		OP01.21–OP01.28, OP02.01–OP02.07, OP02.12, 64002B, 64005B, 65067B, 65068B, 83004B, 83005B, 83011B, 83012B, 83013C, 83015C, 83016B, 83036C, 88037B, 83039B, 83047B, 83077B, 83078B
Ventriculostomy with or without shunting		OP02.2–OP02.4, 83013C, 83051B, 83049B, 83050B, 83052C, 83055B
Removal of subdural/epidural/brain hematoma		OP01.01–OP02.12, 29001C, 29024B, 64143B, 64204B–64206B, 65060B, 83010B, 83013C, 83016B–83019B, 88037B, 83037C, 83038C, 83039B, 83047B, 83056B, 83080B–83082B, 83088B, 84034B
Endarterectomy		OP38.12, 69004B
Bypass surgeries		OP39.28, 69008B, 83063B
**Occupational therapy**	OT	OP93.83
Simple OT		43001A, 43002B, 43003C
Moderate OT		43004A, 43005B, 43006C, 43007A, 43008B, 43009C, 43027C, 43028C
Complicated OT		43029A, 43030B, 43031, 43032C
**Physical therapy**	PT	OP91.1–OP93.6
Simple PT		42001A, 42002B, 42003C, 42004A, 42005B, 42006C
Moderate PT		42007A, 42008B, 42009C, 42010A, 42011B, 42012C, 42017C, 42018C
Complicated PT		42013A, 42014B, 42015C, 42019C
Pharmacological treatment		
Antiepileptics		N03A
Osmotic diuresis		B05BC
**Minor trauma**		Injury severity score (ISS) < 16
**Major trauma**		ISS ≥ 16
**Excluding:**		
Epidemic vertigo		078.81; A88.1
Benign neoplasm of cranial nerves		225.1; D33.3
Vertiginous syndromes and other disorders of vestibular system		386; H81.1–H81.3
Sudden hearing loss		388.2; H91.2
Dizziness and giddiness		780.4; R42
**Events:** Migraine		346; G43; Diagnosis by otolaryngologist or neurologist and medical visits ≧3
Migraine with aura		346.0; G43.1
Migraine without aura		346.1; G43.0
**Comorbidities:**		
Hypertension	HTN	401–405; I10–I15
Diabetes mellitus	DM	250; E10–E14
Depression		296.2, 296.3, 296.82, 300.4, 311; F32, F33, F34.1
Congestive heart failure	CHF	428; I50
Cerebrovascular accident	CVA	430–438; I60–I69
Chronic obstructive pulmonary disease	COPD	490–496; J40–J47
Liver disease		571; K70-K77excluding K70.9
Alcoholism		291, 303, 571.3; F10, K70.9
Chronic kidney disease	CKD	585-586; N18–N19
Gout		274; M10
Osteoporosis		733.0; M81
Hyperlipidemia		272.0–272.4; E78.4, E78.5, E78.8, E78.9
Autoimmune disease	AID	710; M32–M35

**Table A2:** Characteristics of the TBI and non-TBI cohorts at the end of follow-up.

TBI	Total		With		Without		*p* Value
Variables	*n*	%	*n*	%	*n*	%	
**Total**	755,490		151,098	20.00	604,392	80.00	
**Migraine**							<0.001
Without	753,530	99.74	150,557	99.64	602,973	99.77	
With	1960	0.26	541	0.36	1419	0.23	
**Sex**							0.999
Male	469,605	62.16	93,921	62.16	375,684	62.16	
Female	285,885	37.84	57,177	37.84	228,708	37.84	
**Age (years)**	51.26 ± 19.91		51.06 ± 19.80		51.31 ± 19.94		<0.001
**Age group (yrs)**							<0.001
18–29	144,333	19.10	28,822	19.08	115,511	19.11	
30–39	148,152	19.61	28,729	19.01	119,423	19.76	
40–49	122,259	16.18	24,603	16.28	97,656	16.16	
50–59	99,888	13.22	20,592	13.63	79,296	13.12	
≧60	240,858	31.88	48,352	32.00	192,506	31.85	
**Insured premium (NT$)**							<0.001
<15,840	739,707	97.91	147,919	97.90	591,788	97.91	
15,841–25,000	11,311	1.50	2471	1.64	8840	1.46	
>25,001	4472	0.59	708	0.47	3764	0.62	
**Hypertension**							<0.001
Without	660,422	87.42	134,100	88.75	526,322	87.08	
With	95,068	12.58	16,998	11.25	78,070	12.92	
**Diabetes mellitus**							<0.001
Without	672,278	88.99	136,373	90.25	535,905	88.67	
With	83,212	11.01	14,725	9.75	68,487	11.33	
**Depression**							0.526
Without	749,334	99.19	149,847	99.17	599,487	99.19	
With	6156	0.81	1251	0.83	4905	0.81	
**Congestive Heart Failure**							<0.001
Without	732,951	97.02	147,573	97.67	585,378	96.85	
With	22,539	2.98	3525	2.33	19,014	3.15	
**Cerebrovascular accident**							0.004
Without	715,208	94.67	142,815	94.52	572,393	94.71	
With	40,282	5.33	8283	5.48	31,999	5.29	
**Chronic Obstructive Pulmonary Disease**							<0.001
Without	717,782	95.01	144,491	95.63	573,291	94.85	
With	37,708	4.99	6607	4.37	31,101	5.15	
**Liver cirrhosis**							<0.001
Without	717,492	94.97	144,204	95.44	573,288	94.85	
With	37,998	5.03	6894	4.56	31,104	5.15	
**Alcoholism**							<0.001
Without	749,859	99.25	149,362	98.85	600,497	99.36	
With	5631	0.75	1736	1.15	3895	0.64	
**Chronic Kidney Disease**							<0.001
Without	716,140	94.79	145,276	96.15	570,864	94.45	
With	39,350	5.21	5822	3.85	33,528	5.55	
**Osteoporosis**							0.428
Without	753,309	99.71	150,647	99.70	602,662	99.71	
With	2181	0.29	451	0.30	1730	0.29	
**Hyperlipidemia**							<0.001
Without	739,645	97.90	148,439	98.24	591,206	97.82	
With	15,845	2.10	2659	1.76	13,186	2.18	
**Autoimmune Disease**							<0.001
Without	753,281	99.71	150,874	99.85	602,407	99.67	
With	2209	0.29	224	0.15	1985	0.33	
**Season**							<0.001
Spring	185,326	24.53	35,908	23.76	149,418	24.72	
Summer	193,581	25.62	38,195	25.28	155,386	25.71	
Autumn	197,234	26.11	40,407	26.74	156,827	25.95	
Winter	179,349	23.74	36,588	24.21	142,761	23.62	
**Location**							<0.001
Northern Taiwan	296,795	39.29	43,135	28.55	253,660	41.97	
Central Taiwan	220,244	29.15	50,436	33.38	169,808	28.10	
Southern Taiwan	193,657	25.63	48,062	31.81	145,595	24.09	
Eastern Taiwan	41,605	5.51	8888	5.88	32,717	5.41	
Outlying islands	3189	0.42	577	0.38	2612	0.43	
**Urbanization level**							<0.001
1 (The highest)	242,751	32.13	38,210	25.29	204,541	33.84	
2	325,348	43.06	62,274	41.21	263,074	43.53	
3	66,913	8.86	16,935	11.21	49,978	8.27	
4 (The lowest)	120,478	15.95	33,679	22.29	86,799	14.36	

**Table A3:** Factors associated with migraine by the end of follow-up in the Cox regression model.

Variables	Crude HR	95% CI (Low)	95% CI (High)	*p* Value	Adjusted HR	95% CI (Low)	95% CI (High)	*p* Value
**TBI**								
Without	Reference				Reference			
With	1.688	1.454	2.006	< 0.001	1.484	1.276	1.724	<0.001
**Sex**								
Male	0.407	0.356	0.466	<0.001	0.393	0.342	0.452	<0.001
Female	Reference				Reference			
**Age group (yrs)**								
18–29	Reference				Reference			
30–39	0.532	0.412	0.689	<0.001	0.542	0.419	0.702	<0.001
40–49	0.596	0.460	0.773	<0.001	0.692	0.531	0.900	<0.001
50–59	0.597	0.462	0.770	<0.001	0.615	0.472	0.803	<0.001
≧60	0.253	0.196	0.326	<0.001	0.242	0.184	0.319	<0.001
**Insured premium * (NT$)**								
<15,840	Reference				Reference			
15,841–25,000	1.131	0.690	1.854	0.726	1.043	0.636	1.711	0.745
>25,001	0.235	0.034	1.673	0.268	0.232	0.033	1.652	0.291
**Hypertension**								
Without	Reference				Reference			
With	0.876	0.741	1.033	0.084	1.076	0.892	1.299	0.109
**Diabetes mellitus**								
Without	Reference				Reference			
With	1.727	1.603	1.882	<0.001	1.247	0.984	1.547	0.063
**Depression**								
Without	Reference				Reference			
With	6.397	4.977	8.220	<0.001	5.497	5.281	6.069	<0.001
**Congestive Heart Failure**								
Without	Reference				Reference			
With	0.277	0.148	0.516	<0.001	0.434	0.232	0.814	<0.001
**Cerebrovascular** **accident**								
Without	Reference				Reference			
With	2.021	1.654	2.469	<0.001	2.902	2.336	3.605	<0.001
**Chronic Obstructive Pulmonary Disease**								
Without	Reference				Reference			
With	1.066	0.819	1.388	0.184	1.602	1.219	2.104	0.001
**Liver cirrhosis**								
Without	Reference				Reference			
With	0.801	0.580	1.110	0.486	1.059	0.759	1.477	0.577
**Alcoholism**								
Without	Reference				Reference			
With	0.958	0.478	1.922	0.597	1.086	0.382	1.596	0.634
**Chronic Kidney Disease**								
Without	Reference				Reference			
With	1.264	1.159	1.443	<0.001	1.408	1.242	1.695	<0.001
**Osteoporosis**								
Without	Reference				Reference			
With	0.507	0.127	2.031	0.811	0.607	0.152	2.441	0.862
**Hyperlipidemia**								
Without	Reference				Reference			
With	1.682	1.277	2.216	<0.001	1.742	1.307	2.322	<0.001
**Autoimmune Disease**								
Without	Reference				Reference			
With	2.214	1.148	4.269	<0.001	1.845	0.965	3.634	0.073
**Season ****								
Spring	Reference				Reference			
Summer	0.859	0.715	1.033	0.186	0.856	0.712	1.029	0.194
Autumn	0.719	0.597	0.866	<0.001	0.713	0.592	0.859	<0.001
Winter	0.915	0.761	1.101	0.293	0.929	0.772	1.118	0.345
**Location**					**Multicollinearity with urbanization level**			
Northern Taiwan	Reference							
Central Taiwan	1.563	1.330	1.837	<0.001				
Southern Taiwan	1.161	0.971	1.390	0.079				
Eastern Taiwan	1.536	1.181	1.997	<0.001				
Outlying islands	3.254	1.675	6.322	<0.001				
**Urbanization level *****								
1 (The highest)	0.554	0.454	0.676	<0.001	0.665	0.533	0.829	<0.001
2	0.851	0.720	1.007	0.056	0.988	0.828	1.179	0.188
3	0.853	0.663	1.097	0.234	0.776	0.602	0.999	0.049
4 (The lowest)	Reference				Reference			
**Level of care**								
Hospital center	0.454	0.380	0.541	<0.001	0.577	0.472	0.705	<0.001
Regional hospital	0.570	0.490	0.662	<0.001	0.626	0.537	0.731	<0.001
District hospital	Reference				Reference			

HR = hazard ratio, CI = confidence interval, Adjusted HR: Adjusted variables listed in the table. * Insured premium levels were used to reflect the insured individual’s socioeconomic status. ** Refer to the season when the injury occurred in both cohorts or the last visit date when the participants did not experience any injury event. *** The urbanization level was defined by population and certain indicators of the city’s level of development.

**Table A4:** Factors associated with the occurrence of migraine stratified by the variables listed in Table 1 using Cox regression analysis.

TBI	With			Without (Reference)			With vs. Without (Reference)			
Stratified	Events	PYs	Rate (Per 10^5^ PYs)	Events	PYs	Rate (Per 10^5^ PYs)	Adjusted HR	95% CI (Low)	95% CI (High)	*p* Value
**Total**	541	1,010,916.25	53.52	1419	4,027,839.97	35.23	1.484	1.276	1.724	<0.001
**Sex**										
Male	199	597,601.83	33.30	554	2,457,518.36	22.54	1.445	1.244	1.678	<0.001
Female	342	413,314.42	82.75	865	1,570,321.61	55.08	1.522	1.324	1.811	<0.001
**Age group (yrs)**										
18–29	91	64,215.20	141.71	152	201,963.33	75.26	1.833	1.578	2.133	<0.001
30–39	97	197,296.94	49.16	290	874,603.74	33.16	1.236	1.068	1.436	<0.001
40–49	117	157,731.04	74.18	283	634,482.35	44.60	1.665	1.432	1.934	<0.001
50–59	125	175,126.11	71.38	321	693,473.11	46.29	1.516	1.304	1.760	<0.001
≧60	111	416,546.96	26.65	373	1,623,317.44	22.98	1.204	1.035	1.398	0.013
**Insured premium (NT$)**										
<15,840	530	989,004.25	53.59	1393	3,944,623.39	35.31	1.483	1.274	1.723	<0.001
15,841–25,000	11	17,911.53	61.41	25	62,678.54	39.89	1.559	1.341	1.811	<0.001
>25,001	0	4000.47	0.00	1	20,538.04	4.87	0.000	-	-	0.994
**Hypertension**										
Without	448	825,842.92	54.25	1133	3,143,631.22	36.04	1.471	1.262	1.709	<0.001
With	93	185,073.33	50.25	286	884,208.75	32.35	1.513	1.302	1.764	<0.001
**Diabetes mellitus**										
Without	467	863,203.95	54.10	1230	3,339,985.71	36.83	1.439	1.235	1.673	<0.001
With	74	147,712.30	50.10	189	687,854.26	27.48	1.752	1.507	2.037	<0.001
**Depression**										
Without	482	997,210.86	48.33	1308	3,979,648.05	32.87	1.430	1.225	1.670	<0.001
With	59	13,705.39	430.49	111	48,191.92	230.33	1.797	1.543	2.074	<0.001
**Congestive Heart Failure**										
Without	537	978,539.19	54.88	1401	3,859,770.22	36.30	1.511	1.278	1.731	<0.001
With	4	32,377.06	12.35	18	168,069.75	10.71	1.258	1.079	1.455	<0.001
**Cerebrovascular accident**										
Without	462	941,570.69	49.07	1231	3,766,339.26	32.68	1.456	1.252	1.694	<0.001
With	79	69,345.56	113.92	188	261,500.71	71.89	1.606	1.412	1.868	<0.001
**Chronic Obstructive Pulmonary Disease**										
Without	505	951,627.52	53.07	1324	3,770,829.55	35.11	1.448	1.232	1.700	<0.001
With	36	59,288.73	60.72	95	257,010.42	36.96	1.586	1.361	1.837	<0.001
**Liver cirrhosis**										
Without	515	951,563.23	54.12	1361	3,815,760.64	35.67	1.475	1.241	1.655	<0.001
With	26	59,353.02	43.81	58	212,079.33	27.35	1.624	1.408	1.927	<0.001
**Alcoholism**										
Without	530	995,286.01	53.25	1412	3,996,191.15	35.33	1.469	1.251	1.646	<0.001
With	11	15,630.24	70.38	7	31,648.82	22.12	3.310	2.847	3.863	<0.001
**Chronic Kidney Disease**										
Without	519	964,562.99	53.81	1394	3,780,041.75	36.88	1.407	1.219	1.614	<0.001
With	22	46,353.26	47.46	25	247,798.22	10.09	4.368	3.760	5.067	<0.001
**Osteoporosis**										
Without	541	1,006,506.97	53.75	1416	4,010,101.47	35.31	1.487	1.279	1.731	<0.001
With	0	4409.28	0.00	3	17,738.50	16.91	0.000	-	-	0.972
**Hyperlipidemia**										
Without	509	980,866.57	51.89	1330	3,876,852.71	34.31	1.480	1.271	1.700	<0.001
With	32	30,049.68	106.49	89	150,987.26	58.95	1.727	1.484	2.012	<0.001
**Autoimmune Disease**										
Without	538	1,008,504.58	53.35	1403	4,007,721.73	35.01	1.456	1.247	1.701	<0.001
With	3	2411.67	124.40	16	20,118.24	79.53	2.335	2.012	2.723	<0.001
**Season**										
Spring	172	229,526.25	74.94	380	935,681.28	40.61	1.802	1.551	2.093	<0.001
Summer	117	255,445.33	45.80	325	1,023,297.87	31.76	1.415	1.218	1.646	<0.001
Autumn	113	290,161.74	38.94	351	1,138,255.24	30.84	1.225	1.055	1.426	<0.001
Winter	139	235,782.93	58.95	363	930,605.58	39.01	1.478	1.272	1.717	<0.001
**Urbanization level**										
1 (The highest)	74	243,266.98	30.42	299	1,239,987.58	24.11	1.212	1.042	1.409	0.007
2	215	423,537.37	50.76	625	1,784,778.60	35.02	1.410	1.211	1.639	<0.001
3	56	107,878.36	51.91	128	344,857.12	37.12	1.351	1.162	1.575	<0.001
4 (The lowest)	196	236,233.54	82.97	367	658,216.67	55.76	1.480	1.270	1.727	<0.001
**Level of care**										
Hospital center	89	246,378.80	36.12	374	1,365,977.28	27.38	1.287	1.105	1.496	<0.001
Regional hospital	213	481,275.26	44.26	600	1,816,767.20	33.03	1.313	1.130	1.529	<0.001
District hospital	239	283,262.19	84.37	445	845,095.49	52.66	1.562	1.344	1.814	<0.001
